# *Toll-like* receptors 2, 4, and 9 expressions over the entire clinical and immunopathological spectrum of American cutaneous leishmaniasis due to *Leishmania* *(V.) braziliensis* and *Leishmania (L.) amazonensis*

**DOI:** 10.1371/journal.pone.0194383

**Published:** 2018-03-15

**Authors:** Marliane Batista Campos, Luciana Vieira do Rêgo Lima, Ana Carolina Stocco de Lima, Thiago Vasconcelos dos Santos, Patrícia Karla Santos Ramos, Claudia Maria de Castro Gomes, Fernando Tobias Silveira

**Affiliations:** 1 Parasitology Department, Evandro Chagas Institute (Surveillance Secretary of Health, Ministry of Health), Ananindeua, Pará State, Brazil; 2 Pathology Department, Medical School of São Paulo University, São Paulo, São Paulo State, Brazil; 3 Tropical Medicine Institute, Federal University of Pará, Belém, Pará State, Brazil; Taibah University, SAUDI ARABIA

## Abstract

*Leishmania (V*.*) braziliensis* and *Leishmania(L*.*) amazonensis* are the most pathogenic agents of American Cutaneous Leishmaniasis in Brazil, causing a wide spectrum of clinical and immunopathological manifestations, including: localized cutaneous leishmaniasis (LCL^DTH+/++^), borderline disseminated cutaneous leishmaniasis (BDCL^DTH±^), anergic diffuse cutaneous leishmaniasis (ADCL^DTH-^), and mucosal leishmaniasis (ML^DTH++++^). It has recently been demonstrated, however, that while *L*. (*V*.) *braziliensis* shows a clear potential to advance the infection from central LCL (a moderate T-cell hypersensitivity form) towards ML (the highest T-cell hypersensitivity pole), *L*. (*L*.) *amazonensis* drives the infection in the opposite direction to ADCL (the lowest T-cell hypersensitivity pole). This study evaluated by immunohistochemistry the expression of *Toll-like* receptors (*TLRs*) 2, 4, and 9 and their relationships with CD4 and CD8 T-cells, and TNF-α, IL-10, and TGF-β cytokines in that disease spectrum. Biopsies of skin and mucosal lesions from 43 patients were examined: 6 cases of ADCL, 5 of BDCL, and 11 of LCL caused by*L*. (*L*.) *amazonensis*; as well as 10 cases of LCL, 4 of BDCL, and 6 of ML caused by*L*. (*V*.) *braziliensis*. CD4^+^ T-cells demonstrated their highest expression in ML and, in contrast, their lowest in ADCL. CD8^+^ T-cells also showed their lowest expression in ADCL as compared to the other forms of the disease. TNF-α^+^showed increased expression from ADCL to ML, while IL-10^+^and TGF-β^+^ showed increased expression in the opposite direction, from ML to ADCL. With regards to *TLR*2, 4, and 9 expressions, strong interactions of *TLR*2 and 4 with clinical forms associated with *L*. (*V*.) *braziliensis* were observed, while *TLR*9, in contrast, showed a strong interaction with clinical forms linked to *L*. (*L*.) *amazonensis*. These findings strongly suggest the ability of *L*. (*V*.) *braziliensis* and *L*. (*L*.) *amazonensis* to interact with those *TLRs* to promote a dichotomous T-cell immune response in ACL.

## Introduction

American cutaneous leishmaniasis (ACL) is a parasitic protozoan disease caused by different species of the genus *Leishmania* that are widely distributed throughout Latin America [1]. At present, there are at least fifteen recognized species of *Leishmania* within the subgenera *Leishmania*, *Viannia*, and *Mundinia* that may give rise to ACL [2]. In Brazil, where seven of these species are found, *Leishmnia (V*.*) braziliensis* and *Leishmania (L*.*) amazonensis*showthe highest pathogenic potential for humans, being responsible for a wide spectrum of disease, including: localized cutaneous leishmaniasis (LCL), borderline disseminated cutaneous leishmaniasis (BDCL), anergic diffuse cutaneous leishmaniasis (ADCL), and mucosal leishmaniasis (ML) [3].

The immunopathogenesis of ACL has gained great interest due to the complex interactions of *Leishmania* species with human T-cell immune responses. There have been recent findings concerning the clinical-immunopathological spectrum of ACL caused by *L*. *(V*.*) braziliensis* and *L*. *(L*.*) amazonensis* that have helped clarify the immunopathogenic capacities of those two *Leishmania* species. It has been shown that *L*. *(V*.*) braziliensis* and *L*. *(L*.*) amazonensis* may produce not only LCL (the most frequent form of the disease occupying the center of that spectrum, with moderate T-cell hypersensitivity), but principally ML andADCL, the most severe forms occupying the extreme pathogenicity poles of that spectrum; i.e., the highest and lowest T-cell hypersensitivity, respectively. Additionally, those *Leishmania* species may also produce BDCL, an intermediary form showing partial inhibition of T-cell hypersensitivity between the central LCL and the two polar forms, ML and ADCL, which can occupy both sides of that spectrum (i.e., BDCL may be produced either by *L*. *(V*.*)* spp. or *L*. *(L*.*)* spp.) [4]. It should also be noted that the immunopathogenic abilities of *L*. *(V*.*) braziliensis* and *L*. *(L*.*) amazonensis* have been confirmed in experimental BALB/c mice model–which have shown that those *Leishmania* species are able to modulate differential expressions of dendritic cells and T-cell immune responses [5].

With regards to the immunopathology of ACL, there is recent evidence of the involvement of CD4^+^ (Th1, Th2, Th17 and Treg-Foxp3+CD4+CD25+) and CD8^+^ T-cell subset profiles, as well as some cytokines produced by those cells, such as, IFN-γ, IL-4, IL-10, and TGF-β, as well as iNOS expression over the entire clinical-immunopathological spectrum of the disease caused by *L*. *(V*.*) braziliensis* and *L*. *(L*.*) amazonensis*. In that respect, the pivotal role of CD4^+^/Th1-type immune response associated to significant expression of CD8^+^ T-cells, IFN-γ, and iNOS should be emphasized in determining resistance against leishmanial infections. That is, in general, the immune response profile found in most clinical forms of ACL (LCL, BDCL and ML)—with exception of ADCL, which is characterized by a strong CD4^+^/Th2-type immune response associated with low levels of CD8^+^ T-cells and high expression of IL-4, IL-10 and TGF-β cytokines. Treg(Foxp3)-cells were also detected at low to moderate levels in most clinical forms of ACL (LCL, BDCL and ML), but at higher levels in ADCL [6].

The essential role of *Toll-like* receptors (*TLRs*) in the development of the innate and adaptive immune responses against different types of invasive microorganisms should not be forgotten. *TLRs* are transmembrane glycoproteins that lend high levels of specificity to innate immune responses by recognizing every type of invasive microorganism that may infect humans. *TLRs* are principally found either within the plasma membrane or within the internal membranes of macrophages, DCs, and NK cells. They may also be found, with lower expression, in T and B lymphocytes [7, 8]. Ten *TLRs* have so far been described in humans (*TLR*1-10) that can be separated into two groups, depending upon their localization within cells: i) *TLR*1, 2, 4, 5 and 6 encountered on the plasmatic surface, with intracellular protein domains of the TIR type (MyD88, TIRAP, TRIF and TRAM), and, ii) *TLR*3, 7, 8 and 9 that reside in internal membranes, such as lysosomes, but without specific extracellular domains; little is currently known about *TLR*10 [9]. Each *TLR* receptor has its own self-signaling pathway that promotes specific biological responses leading to the sensitization of genes involved in host defenses against microorganisms. Thus, after recognition of a specific antigen, *TLRs* trigger NF-κB to reach the nucleus, allowing the transcription and production of pro-inflammatory cytokines. This process usually requires the intervention of an adaptor protein having the TIR repeated domain, with MyD88 being the molecule most commonly used by *TLRs* (with exception of *TLR*3) [10].

In terms of *Leishmania* sp. infections, there is *in vitro* and *in vivo* evidence demonstrating the crucial role of *TLRs* in the development of protective immune responses against those infections, and recent studies have largely concentrated on *TLR*2, 4, and 9 [11]. Additionally, the indispensable role ofMyD88 in the generation of protective immunity to *L*. *(V*.*) braziliensis*-infections in mice should be mentioned here, with *TLR*2 appearing to have a regulatory role during infection [12]. When *TLR*2 and 4 expressions were investigated in LCL caused by the same *Leishmania* species, however, a higher expression of *TLR*2 as compared to that of *TLR*4 was observed during the active disease (mainly in macrophages)—although without correlations with cytokine titers or number of cells [13]. The role of *TLR*2 was also examined in two different clinical forms of ACL (LCL and ADCL) caused by *L*. *(L*.*) mexicana* in Mexico [14, 15]–a parasite closely related to *L*. *(L*.*) amazonensis* in Brazil [4]. In the first approach, the ability of *TLR*2 ligands to restore the effector function of exhausted CD8^+^ T-cells from ADCL patients against *L*. *(L*.*) mexicana*-infected macrophages was demonstrated [14]. In the second approach, a higher down-regulation of the innate immune response-genes in NK cells from severe ADCL as compared to those from LCL, especially those related to *TLR*2 and JAK/STAT signaling pathways, was confirmed [15]. The role of *TLR*9 was also investigated in LCL due to *L*. *(V*.*) braziliensis* showing a strong association with granuloma in the dermis of cutaneous lesions of patients, principally in macrophage cells [16]. However, although *TLR*9 was associated with granuloma in the dermis of cutaneous lesions of patients with LCL caused by *L*. *(V*.*) braziliensis*, experiments with *TLR*9_/_mice infected with the same parasite species showed that *TLR*9 signaling is important for early control of lesion development and parasite burdens, but is dispensable for the differentiation of Th1 cells secreting IFN-γ [17]. In other experiments regarding the roles of MyD88- or *TLR*9-dependent signaling pathways in mice infected by *L*. *(V*.*) guyanensis*, the principal role of both MyD88 and *TLR*9 in the development of Th1-dependent healing responses against *L*. *(V*.*) guyanensis* was demonstrated [18].

Thus, taking into account the above comments, we decided to investigate *TLR*2, 4, and 9 expressions over theentire clinical-immunopathological spectrum of ACL caused by *L*. *(V*.*) braziliensis* and *L*. *(L*.*) amazonensis* to better understand the immunopathogenesis of the disease. The present results provided strong evidence for associating *TLR*2 and 4 with beneficial T-cell immune response in LCL and BDCL caused either by *L*. *(V*.*) braziliensis* or *L*. *(L*.*) amazonensis*. In contrast, *TLR*9 showed strong association with the inhibition of T-cell immune responses in ACL forms caused by *L*. *(L*.*) amazonensis*, principally the subpolar BDCL and polar ADCL forms.

## Materials and methods

### Study design

The Present study was of the cross-sectional type, in which we analyzed skin and/or mucosal samples (biopsies) from patients with ACL caused by *L*. *(V*.*) braziliensis* (*L*.*b*) and *L*. *(L*.*) amazonensis* (*L*.*a*) by immunohistochemistry for the expression of *TLR*2, 4, and 9 as well as CD4 and CD8 T-cells and the cytokines TNF-α, IL-10 and TGF-β in the immunopathogenesis of the different clinical forms of that disease (LCL/*L*.*b*-*L*.*a*, BDCL/*L*.*b*-*L*.*a*, ML/*L*.*b* and ADCL/*L*.*a*).

### Clinical samples

Biopsies of skin and mucosal lesions from 43 patients were examined: six cases of ADCL, five of BDCL, and eleven of LCL caused by *L*. *(L*.*) amazonensis*; ten cases of LCL, four of BDCL, and six of ML caused by *L*. *(V*.*) braziliensis*. Other data on age, gender, time and the type of lesion, and the DTH skin reaction of the patients are presented in Table 1. Patients diagnosed parasitologically and/or immunologically (DTH) with ACL and treatment-naïve were selected for the present study. The patients were examined in the ambulatory of the “Prof. Dr. Ralph Lainson Leishmaniasis Laboratory” at the Evandro Chagas Institute (SVS-MS), Ananindeua, Pará State, Brazil, during the period between January/2012 and December/2015. All of the patients were submitted to clinical and laboratory evaluations for ACL diagnosis and all of the confirmed clinical cases received specific treatments for that disease. However, as all of the six ADCL patients had been unsuccessfully treated for more than ten years in our ambulatory, all had already experienced different therapeutic schemes with meglumine antimoniate, pentamidine isetionate, or Amphotericin B. All of the clinical and laboratory procedures to confirm ACL diagnosis were performed as described in earlier works [19, 20].

**Table 1:** Individual and clinical-immunological characteristics of the patients suffering from ACL caused by *L*. *(V*.*) braziliensis* and *L*. *(L*.*) amazonensis*.

Clinical forms	Gender		Age (years)	Time of lesion*	Type of lesion	DTH skin reaction
	Male	Female				
ADCL/*L*.*a*	5	1	24–59	154–420	Infiltrative/nodular	Negative
BDCL/L.a	5	0	24–49	11–60	Infiltrative	Negative
LCL/ *L*.*a*	8	2	19–66	2–6	Ulcerated	Negative
LCL/ *L*.*b*	10	2	26–46	2–5	Ulcerated	Positive
BDCL/ *L*.*b*	3	1	34–48	1–4	Equithymatoid	Positive
ML/*L*.*b*	6	0	27–71	34–120	Ulcerated	Positive

*Time of lesion: *months*

ADCL/*L*.*a*: anergic diffuse cutaneous leishmaniasis by *L*. *(L*.*) amazonensis*;

BDCL/*L*.*a*: borderline disseminated cutaneous leishmaniasis by *L*. *(L*.*) amazonensis*;

LCL/*L*.*a*: localized cutaneous leishmaniasis by *L*. *(L*.*) amazonensis*;

LCL/*L*.*b*: localized cutaneous leishmaniasis by *L*. *(V*.*) braziliensis*;

BDCL/*L*.*b*: borderline disseminated cutaneous leishmaniasis by *L*. *(V*.*) braziliensis*;

ML/*L*.*b*: mucosal leishmaniasis by *L*. *(V*.*) braziliensis*.

### Isolation and identification of *L*. *(V*.*) braziliensis* and *L*. *(L*.*) amazonensis* from cutaneous and mucosal lesions of the patients

The process for isolating *Leishmania* spp. from patients suffering from ACL was published previously [19, 20]. The characterization of *Leishmania* species was performed using PCR-RFLP molecular techniques that utilized two target sequences: one of the RNA polymerase II gene, in which products of the PCR amplifications using RPOF2 and RPOR2 primers (Coralville, IOWA, USA) were cleaved using TspRI and HgaI restriction enzymes (New England Biolabs—Ipswich, Massachusetts, USA), and another of the hsp70 gene, whose products were purified and cleaved using HaeIII restriction enzyme (Invitrogen—Carsbad, Califórnia, USA); both products were used to detect polymorphisms and then compared with reference strains of the subgenera *Viannia* and *Leishmania* known to act as ACL agents in northern Brazil [21–23].

### Immunohistochemical analyses

*Processing*: A fragment of each biopsy was fixed in 10% neutral formaldehyde and embedded in paraffin. Histological sections (4 μm) were made and collected on biotinylated slides (SIGMA), diaphonized in two baths of cold xylene (20 and 10 minutes each), and then hydrated in a decreasing ethanol series (100%, 90%, 80% and 70%) lasting 2 min. each, being subsequently immersed in a pH 7.4 PBS (Phosphate Buffered Saline) solution for 5 min.

### Immunohistochemical reactions

This process was undertaken following a previously described technique, with modifications. We used the same immunohistochemical protocol for the immunological markers in the present study, except for the *TLRs*, which were performed during differential steps designed for double marking (varying only in the primary antibody step, its dilution, and antigenic recuperation). We employed the labeled Streptavidin-biotin method, LSAB-HRP (Dako—Carpinteria, California, USA), in which a secondary biotinylated antibody reacts with various molecules of streptavidin conjugated to peroxidase [3, 24]. S1 Table lists the primary antibodies used, their codes, dilutions, and the treatments utilized for antigen exposition.

### Immunohistochemical staining of CD4 and CD8 T-cells

Incubations of the histological sections against CD4 and CD8 T-cells diluted in PBS with 1% BSA overnight at 4 °C were performed under the same conditions described above for the primary antibodies (S1 Table) [3].

### Immunostaining of TNF-α, IL-10 and TGF-βcytokines

After removing the paraffin, hydrating the tissue sections, blocking endogenous peroxidase, and antigen recuperation using 10 mM citrate buffer (pH 6.0), as described above, the slides were washed in distilled water for 5 min. and then in PBS buffer containing 0.1% saponin for 10 min., being subsequently washed in distilled water and PBS (pH 7.4) for 5 min. each. Nonspecific tissue proteins were then blocked by incubation in a 10% skimmed milk solution (Molico^®^, Nestle) for 30 min. at room temperature. The slides were subsequently incubated overnight (at 4 °C) with the primary antibodies anti-TNF-α, anti-IL-10, and anti-TGF-β diluted in 1% BSA. The application reaction was performed using a LSAB-HRP kit (Dako—Carpinteria, California, USA), and the visualization reactions as well as subsequent steps were performed as previously described [24].

### Double immunohistochemical staining for CD68 macrophages expressing *TLR*2, 4, and 9

Double immunohistochemical staining was used to verify the expression of *TLR*2, 4, and 9 on CD68+ macrophages. The protocol utilized for this reaction followed Tuon et al. [16], with some adaptations. These reactions are based on the same steps as the conventional immunohistochemical reactions already described, with few modifications. To investigate CD68+ macrophages, antigen exposure was performed in a Pascal pan at 95 °C for 20 min. in 10 mM citrate buffer (pH 6.0). The slides were then maintained at room temperature for 20 min. followed by the immunohistochemical protocol and visualization with DAB.

After visualization, in a second step, the sections were washed in distilled water and PBS (pH 7.4) for 5 min. each, and then incubated with goat primary anti-*TLR*2 (SC 8689), anti-*TLR*4 (SC 8694), and anti-*TLR*9 (SC 13215) antibodies for 12 hours at 4 °C. The following step involved the exposure of the sections to the reagents of the LSAB + System-AP kit (Dako—Carpinteria, California, USA, code K0689) conjugated with a ready-to-use polymer of alkaline phosphatase in a humid chamber for 30 min. at 37 °C. The slides were subsequently washed in PBS (pH 7.4) and the linkage sites visualized with Liquid Permanent Red(Dako—Carpinteria, California, USA, code K0640).

The sections were subsequently washed in distilled water and PBS, stained with hematoxylin Harris for proximately 1 min., washed in distilled water, dehydrated in an ethanol series (70%, 80%, 90%, and absolute), diaphonized in xylene, and left to dry at room temperature. The sections were then mounted in Entellan resin.

### Determining cell immunostaining density

In analyzing the immunostaining of the different cell phenotypes and cells expressing cytokines, the brown staining of their cytoplasm was considered a positive identification. Double immunohistochemical staining considered macrophage cells showing brown staining with co-localized *TLR* stained red as demonstrating *TLR* expression [16].

The immunostained cells were counted using an image analysis system (Axioskop 2 plus Zeiss) coupled to a microcomputer running the AxioVision 4.0 program. We photographed 10 fields of each histological section under a 40x objective, and the immunostained cells were counted using Imaje J software [25]. The mean numbers of marked cells per field were calculated and cell population densities were determined from the ratios of the marked cells per area (μm^2^), which are presented here in mm^2^.

### Ethics approval

This study was approved by the Ethics Committee in Human Research of the Evandro Chagas Institute (Surveillance Secretary of Health, Ministry of Health, Brazil) and Brazil Platform, under protocol number 102.885/2012. All patients enrolled in this study were informed about the study and signed a free-consent form in accordance with the principles of the Declaration of Helsinki.

### Statistical analysis

The data were expressed as the means ± standard deviations. The parametric ANOVA test with the Tukey post-test was used to compare the quantitative values of each of the cell phenotypes (CD4 and CD8 T-cells), cytokines (TNF-α, TGF-β and IL-10), and the *TLR*2, 4, and 9 of the different clinical forms of ACL caused by *L*. *(V*.*) braziliensis* or *L*. *(L*.*) amazonensis*. The correlations of the expression of *TLR*2, 4, and 9 with the expression of CD4^+^ and CD8^+^ T-cells, as well as the cytokines (TNF-α^+^, TGF-β^+^ and IL-10^+^) were performed using the Pearson correlation index. The results were considered significant when the probability of a significant error was less than 0.05 (p< 0.05). All of the statistical procedures were performed using Graph Pad Prism version 4.0 for Windows software (Graph Pad Software, San Diego, CA).

## Results

### CD4^+^ and CD8^+^ T-cells densities among different clinical forms of ACL caused by *L*. *(V*.*) braziliensis* and *L*. *(L*.*) amazonensis*

With regards to CD4^+^ T-cell densities in the relative spectrum of ACL caused by *L*. *(L*.*) amazonensis*, there were no differences (p> 0.05) between the central form LCL (1465 ± 194.3 cells/mm^2^) and the subpolar BDCL form (1198 ± 84.2 cells/mm^2^), although both clinical forms showed higher cell densities (p< 0.05) than the polar form ADCL (394 ± 159.3 cells/mm^2^): LCL/BDCL > ADCL. It is also important to note that the ADCL form showed the lowest CD4^+^ T-cell density (p< 0.05) when compared to the other clinical forms of ACL. When the CD4^+^ T-cell densities were examined in terms of the relative spectrum of ACL caused by *L*. *(V*.*) braziliensis*, we observed a progressive and significant increase (p< 0.05) starting with the central LCL form (736 ± 134.9 cells/mm^2^) towards the BDCL form (1696 ± 105.6 cells/mm^2^) up to the ML form (2068 ± 245.0 cells/mm^2^), with significant differences (p< 0.05) between them: LCL < BDCL < ML. As such, it could be observed that CD4^+^ T-cell density in the ML form was greater (p< 0.05) than any of the other clinical forms of ACL, whether caused by *L*. *(V*.*) braziliensis* (LCL and BDCL) or by *L*. *(L*.*) amazonensis* (LCL, BDCL and ADCL) (S1 Fig).

With regards to CD8^+^ T-cell densities in the relative spectrum of ACL caused by *L*. *(L*.*) amazonensis*, as observed in terms of CD4^+^ T-cell densities, there were no differences (p> 0.05) between the central LCL form (1781 ± 133.0 cells/mm^2^) and the subpolar BDCL form (1529 ± 175.1 cells/mm^2^), even though both demonstrated cell densities greater (p< 0.05) than the polar form ADCL (816 ± 231.0 cells/mm^2^): LCL/BDCL > ADCL. It was also observed that the ADCL form demonstrated lower densities of CD8^+^ T-cell (p< 0.05) as compared to the other clinical forms of ACL. As such, and considering the relative spectrum of ACL caused by *L*. *(V*.*) braziliensis*, an equivalence (p> 0.05) could be observed between the cell densities of the central LCL (1568 ± cells/mm^2^) and polar ML (1741± 175.5 cells/mm^2^) forms, although only the cell density of ML form was seen to be greater than the BDCL (1364 ± 179.6 cells/mm^2^) form (p< 0.05). It must also be noted that when examining the CD8^+^ T-cell densities within the complete spectrum of ACL, the cell density of the LCL/*L*.*a* form (1781 ± 133.0 cells/mm^2^) was greater (p< 0.05) than that of the BDCL/*L*.*b* form (1364 ± 179.6 cells/mm^2^) (S2 Fig).

### Comparison of CD4^+^ and CD8^+^ T-cells densities in the different clinical forms of ACL caused by *L*. *(V*.*) braziliensis* and *L*. *(L*.*) amazonensis*

**Fig 1:**
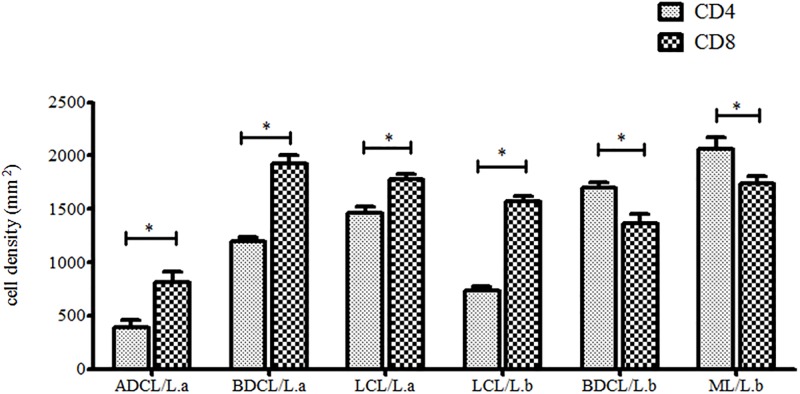
CD4^+^ and CD8^+^ T-cell densities in the different clinical forms of ACL caused by *L*. (*V*.) *braziliensis* (*L*.*b*)and *L*. (*L*.) *amazonensis* (*L*.*a*). (*) = p< 0.05.

Comparative analyses clearly showed that there were higher expressions of CD8^+^ T-cells throughout most of ACL spectrum (p< 0.05), including the two central LCL forms caused by *L*. *(V*.*) braziliensis* or *L*. *(L*.*) amazonensis*, in addition to the BDCL and ADCL forms caused by *L*. *(L*.*) amazonensis*. Only the BDCL and ML forms caused by *L*. *(V*.*) braziliensis* showed higher (p< 0.05) CD4^+^ T-cell expressions (Fig 1).

### Cell densities expressing TNF-α, TGF-β and IL-10 among different clinical forms of ACL caused by *L*. *(V*.*) braziliensis* and *L*. *(L*.*) amazonensis*

**Fig 2:**
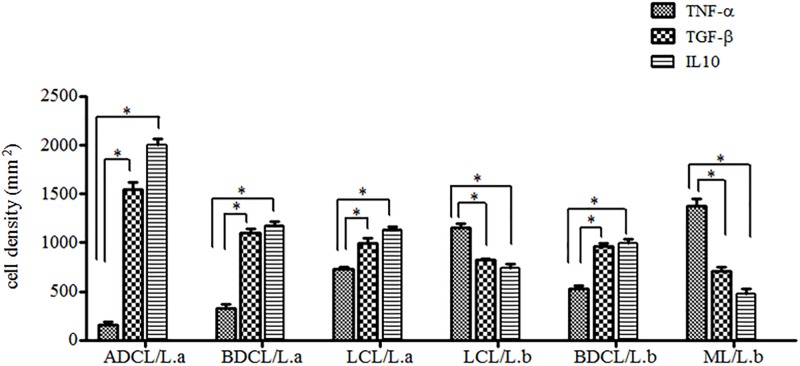
Comparison of cell densities expressing TNF-α^+^, TGF-β^+^ and IL-10^+^ in the different clinical forms of ACL caused by *L*. (*V*.) *braziliensis* (*L*.*b*)and *L*. (*L*.) *amazonensis* (*L*.*a*). (*) = p< 0.05.

This analysis refers to the spectrum of ACL caused by *L*. *(L*.*) amazonensis*, which revealed TNF-α^+^ cell densities of the central LCL form (732 ± 67.0 cells/mm^2^) greater (p< 0.05) than those of the subpolar BDCL (330 ± 80.4 cells/mm^2^) and polar ADCL (162 ± 54.5 cells/mm^2^) forms, with the latter two having equivalent cell densities (p> 0.05). In terms of the relative spectrum of ACL caused by *L*. *(V*.*) braziliensis*, however, it was observed that TNF-α^+^ cell densities in the polar ML form (1376 ± 176.7 cells/mm^2^) were greater (p< 0.05) than the two other forms having the same etiology (LCL [1154 ± 134.0 cells/mm^2^] and BDCL [532 ± 65.0 cells/mm^2^]), and were also greater (p< 0.05) than the forms of the disease caused by *L*. *(L*.*) amazonensis*: LCL, BDCL, and ADCL. It should also be noted that TNF-α^+^ cell densities in the LCL form (1154 ± 134.0 cells/mm^2^) caused by *L*. *(V*.*) braziliensis* were greater (p< 0.05) than the BDCL form (532 ± 65.0 cells/mm^2^) caused by that same species, as well as those of the LCL, BDCL and ADCL forms caused by *L*. *(L*.*) amazonensis* (S3 Fig)-(Fig 2).

This analysis of the spectrum of ACL caused by *L*. *(L*.*) amazonensis* showed that the TGF-β^+^ cell densities of the polar ADCL form (1595 ± 189.1 cells/mm^2^) were not only greater than (p< 0.05) the subpolar BDCL (1098 ± 96.5 cells/mm^2^) and central LCL (991 ± 165.0 cells/mm^2^) forms, but were also greater than the three forms of the spectrum of *L*. *(V*.*) braziliensis*: the central LCL (820 ± 69.1 cells/mm^2^), subpolar BDCL (958 ± 67.9 cells/mm^2^), and polar ML (708 ± 98.8 cells/mm^2^) forms. As such, it can be concluded that the polar ADCL/*L*.*a* form shows greater (p< 0.05) TGF-β^+^ cell density than those of the other clinical forms of ACL. Additionally, it should be noted that although there were no significant differences (p> 0.05) between the other two clinical forms caused by *L*. *(L*.*) amazonensis* (the subpolar BDCL [1098 ± 96.5 cells/mm^2^] and central LCL [991 ± 165.0 cells/mm^2^]) forms, both demonstrated greater expressions (p< 0.05) than the centralLCL (820 ± 69.1 cells/mm^2^) and polar ML (708 ± 98.8 cells/mm^2^) forms caused by *L*. *(V*.*) braziliensis*. Finally, it should also be observed that the subpolar BDCL form (958 ± 67.9 cells/mm^2^) caused by *L*. *(V*.*) braziliensis* demonstrated a greater TGF-β^+^ celldensity (p< 0.05) than the neighboring polar ML form (708 ± 98.8 cells/mm^2^) (S4 Fig)-(Fig 2).

In relation to the clinical forms of ACL caused by *L*. *(L*.*) amazonensis*, the profiles of immunostained cell densities (IL-10^+^) were very similar to those of TGF-β^+^; that is, the polar form ADCL (1945± 160.2 cells/mm^2^) demonstrated greater expression (p< 0.05) than the neighboring subpolar BDCL (1172 ± 79.2 cells/mm^2^) and central LCL(1136 ± 100.0 cells/mm^2^) forms, as well as greater expression than the three other forms caused by *L*. *(V*.*) braziliensis* (central LCL [735 ± 150.9 cells/mm^2^], subpolar BDCL [992 ± 117.1 cells/mm^2^], and polar ML [479 ± 117 cells/mm^2^]). It can therefore be concluded that, in the same way as seen with TGF-β^+^, the ADCL form showed the greatest (p< 0.05) IL-10^+^ cell density when compared to the other clinical forms of ACL. It was also observed that, while no significant differences (p> 0.05) were noted between the BDCL (1172 ± 79.2 cells/mm^2^) and LCL(1136 ± 100.0 cells/mm^2^) forms caused by *L*. *(L*.*) amazonensis*, both demonstrated greater (p< 0.05) IL-10^+^ cell densities than seen in the LCL (735 ± 150.9 cells/mm^2^) and ML (479 ± 117 cells/mm^2^) forms caused by *L*. *(V*.*) braziliensis*. However, the BDCL (992 ± 117.1 cells/mm^2^) form caused by *L*. *(V*.*) braziliensis* showed greater (p< 0.05) IL-10^+^ cell densities than the congenerous LCL (735 ± 150.9 cells/mm^2^) and ML (479 ± 117 cells/mm^2^) forms. Lastly, it is important to emphasize that the ML/*L*.*b* form demonstrated the lowest (p< 0.05) IL-10^+^ cell density among all of the clinical forms of ACL (S5 Fig)-(Fig 2).

### Comparisons of cell densities expressing TNF-α, TGF-β, and IL-10 in the different clinical forms of ACL caused by *L*. *(V*.*) braziliensis* and *L*. *(L*.*) amazonensis*

Comparative analyses of cell densities expressing TNF-α^+^, TGF-β^+^, and IL-10^+^ in the different clinical forms of ACL caused by *L*. *(V*.*) braziliensis* and *L*. *(L*.*) amazonensis* showed greater (p< 0.05) expressions of TGF-β^+^ andIL-10^+^ cytokines in the clinical forms associated with *L*. *(L*.*) amazonensis* (such as LCL, BDCL and ADCL), while TNF-α^+^ demonstrated greater (p< 0.05) expression in the clinical forms associated with *L*. *(V*.*) braziliensis*(principallyLCL and ML) (Fig 2).

### Macrophage (CD68^+^) cell densities expressing *TLR*2^+^, 4^+^ and 9^+^ in the different clinical forms of ACL caused by *L*. *(V*.*) braziliensis* and *L*. *(L*.*) amazonensis*

**Fig 3:**
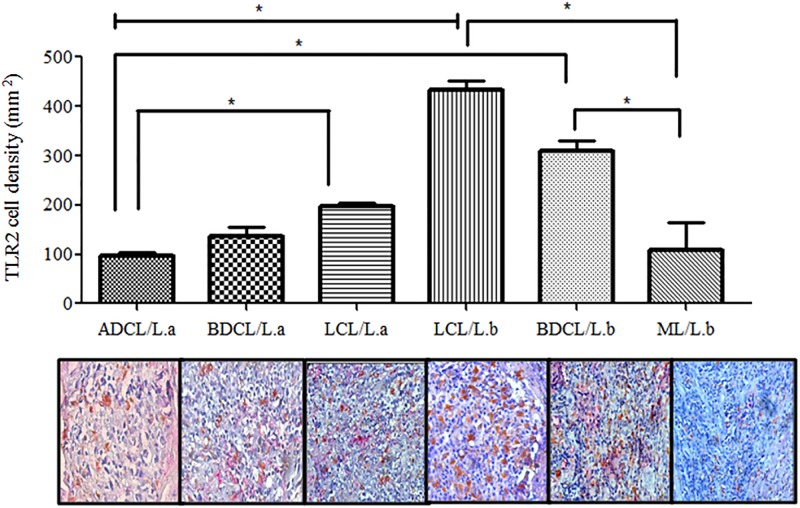
CD68^+^ macrophage cell densities expressing *TLR*2^+^ in the different clinical forms of ACL caused by *L*. (*V*.) *braziliensis (L*.*b)* and *L*. *(L*.*) amazonensis(L*.*a)*. (*) = p< 0.05. (⊢⊣) = significant differences between the clinical forms, localized cutaneous leishmaniasis LCL/*L*.*b* x LCL/*L*.*a*, borderline disseminated cutaneous leishmaniasis (BDCL/*L*.*a*), and anergic diffuse cutaneous leishmaniasis (ADCL/*L*.*a*).

In terms of the relative spectrum of ACL caused by *L*. *(L*.*) amazonensis*, no differences were observed (p> 0.05) in *TLR*2^+^ expression between the central LCL (197 ± 24.6 cells/mm^2^) and subpolar BDCL (136 ± 45.4 cells/mm^2^) forms, with only the central LCLform showing greater expression (p< 0.05) than the polar ADCL form (96 ± 19.2 cells/mm^2^). In terms of the clinical forms caused by *L*. *(V*.*) braziliensis*, on the other hand, it was observed that while the central LCL (433 ± 102 cells/mm^2^) and subpolar BDCL (310 ± 41.9 cells/mm^2^) forms demonstrated no significant differences (p> 0.05) in their *TLR*2^+^ expressions, both forms showed greater expressions (p< 0.05) than the polar MLform (110 ± 133.0 cells/mm^2^). More significant than this, however, was the fact that the central LCL/*L*.*b* form showed greater expression (p< 0.05) than any of the forms caused by *L*. *(L*.*) amazonensis* (LCL, BDCL, and ADCL), while the subpolar BDCL/*L*.*b* form was only greater (p< 0.05) than the ADCL form (Fig 3).

**Fig 4:**
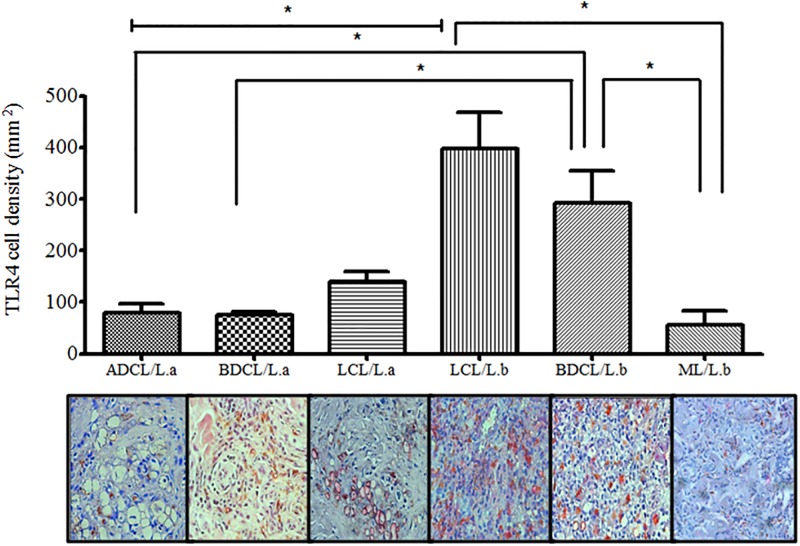
CD68^+^ macrophage cell densities expressing *TLR*4^+^ in the different clinical forms of ACL caused by *L*. (*V*.) *braziliensis* (*L*.*b*)and *L*. *(L*.*) amazonensis* (*L*.*a*). (*) p < 0.05; (⊢⊣): significant differences between clinical forms, localized cutaneous leishmaniasis (LCL/*L*.*b* x LCL/*L*.*a)*, borderline disseminated cutaneous leishmaniasis (BDCL/*L*.*a*), and anergic diffuse cutaneous leishmaniasis (ADCL/*L*.*a*).

Analyses of macrophage (CD68^+^) cell densities expressing *TLR*4^+^in the different clinical forms of ACL revealed expression profiles very similar to those of *TLR*2^+^, showing only some differences in the relative spectrum of ACL caused by *L*. *(L*.*) amazonensis*, with no differences (p> 0.05) among the forms LCL (141 ± 60.1 cells/mm^2^), BDCL (76 ± 14.9 cells/mm^2^), and ADCL (79 ± 45.8 cells/mm^2^). In terms of the spectrum of ACL caused by *L*. *(V*.*) braziliensis*, the only observation to be added here refers to the fact that the subpolar form BDCL (292 ± 125.8 cells/mm^2^) showed a higher expression (p< 0.05) than both the polar ADCL (79 ± 45.8 cells/mm^2^) and the subpolar BDCL (76 ± 14.9 cells/mm^2^) forms, both caused by *L*. *(L*.*) amazonensis*. Additionally, there were no differences (p> 0.05) in expression between the LCL/*L*.*b* (399 ± 140 cells/mm^2^) and BDCL/*L*.*b* (292 ± 125.8 cells/mm^2^) forms, although both were greater (p< 0.05) than the polar ML/*L*.*b* form (56 ± 66.1 cells/mm^2^) (Fig 4).

**Fig 5:**
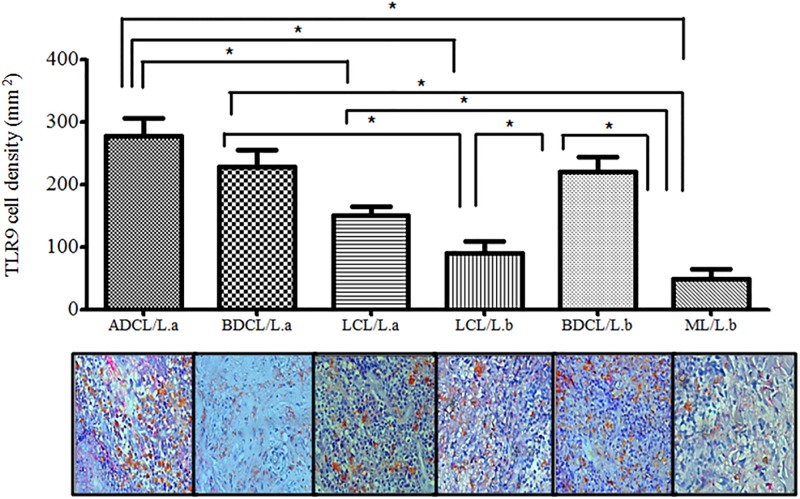
CD68^+^ macrophage cell density expressing *TLR*9^+^ in the different clinical forms of ACL caused by *L*. (*V*.) *braziliensis* (*L*.*b*)and *L*. (*L*.) *amazonensis* (*L*.*a*). (*) = p< 0.05.

In terms of the analysis of the spectrum of ACL caused by *L*. *(L*.*) amazonensis*, the polar ADCL form (277 ± 70.4 cells/mm^2^) demonstrated greater *TLR*9^+^ expression (p< 0.05) than the central LCL form (150 ± 48.7 cells/mm^2^), although no differences were observed (p> 0.05) between the polar ADCL and the subpolar BDCLforms (228 ± 60.1 cells/mm^2^), which likewise did not demonstrate any significant differences (p> 0.05) when compared to the central LCL form. Additionally, when comparing the spectrum of ACL caused by *L*. *(V*.*) braziliensis*, the subpolar BDCL form (221 ± 47.5 cells/mm^2^) demonstrated greater *TLR*9^+^ expression (p< 0.05) than either the LCL (90 ± 67.9 cells/mm^2^) or ML (48 ± 38.6 cells/mm^2^) forms; no significant differences were observed (p> 0.05) between the latter two forms (LCLandML) (Fig 5).

### Comparison of macrophage (CD68^+^) cell densities expressing *TLR*2, 4, and 9 in the different clinical forms of ACL caused by *L*. *(V*.*) braziliensis* and *L*. *(L*.*) amazonensis*

**Fig 6:**
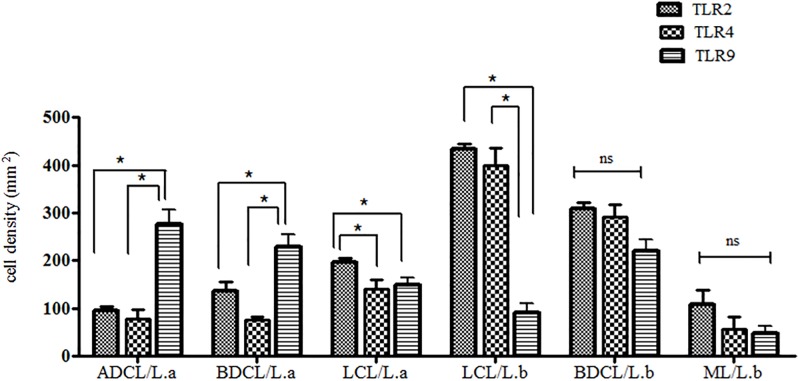
*TLR*2^+^, 4^+^, and 9^+^ expressions in the different clinical forms of ACL caused by *L*. (*V*.) *braziliensis* (*L*.*b*)and *L*. (*L*.) *amazonensis* (*L*.*a*). (*) = p< 0.05.

Comparatively, we found stronger interactions of *TLR*2^+^ and 4^+^ with clinical infection by *L*. *(V*.*) braziliensis*, with greater expressions of those receptors in the LCL and BDCL forms, but weaker interactions with clinical infection by *L*. *(L*.*) amazonensis*, with only modest expression in the LCL and BDCL forms. In terms of *TLR*9^+^ expression, we identified stronger interactions with clinical infection by *L*. *(L*.*) amazonensis* as opposed to the expressions of *TLR*2^+^ and 4^+^, indicated by greater expression in the ADCL/*L*.*a* and BDCL/*L*.*a* forms; significant expression of *TLR*9^+^ in the BDCL form caused by *L*. *(V*.*) braziliensis* was also noted (Fig 6).

### Correlations of *TLR*2^+^, 4^+^, and 9^+^ with CD4^+^ and CD8^+^ T-cells and TNF-α^+^, TGF-β^+^, and IL-10^+^ cytokine expressions

**Fig 7:**
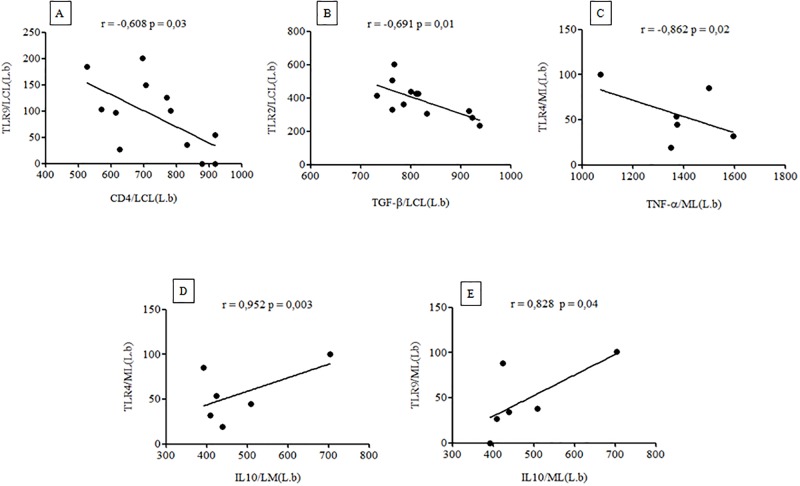
Correlations of *TLR*2^+^, 4^+^, and 9^+^ with CD4^+^/CD8^+^ T-cells and TNF-α^+^, TGF-β^+^, and IL-10^+^ cytokine expressions in the LCL and ML clinical forms of ACL caused by *L*. *(V*.*) braziliensis*. (A) *TLR* 9^+^ X CD4^+^; (B) *TLR* 2^+^ X TGF-β^+^; (C) *TLR* 4^+^ X TNF-α^+^; (D) *TLR* 4^+^ X IL-10^+^; (E) *TLR* 9^+^ X IL-10^+^.

Considering that most of the analyses did not reveal any correlations (and those cases that did were generally without statistical significance [p> 0.05]), the following results will be restricted to only five cases that showed significant correlations (p< 0.05)–all of them related to clinical forms caused only by *L*. *(V*.*) braziliensis*: 1) *TLR9*^+^ *versus CD4*^+^ *T-cells*: a significant negative correlation was seen only in the LCLform (*r* = -0.608 p = 0.03) (Fig 7A); 2) *TLR2*^+^ *versus TGF-β*^+^: a significant negative correlation was only seen in the LCL form (*r* = -0.691 p = 0.01) (Fig 7B); 3) *TLR4*^+^ *versus TNF-α*^+^: a significant negative correlation was only seen in the ML form (*r* = -0.862 p = 0.02) (Fig 7C); 4) *TLR4*^+^ *versus IL-10*^+^: a significant positive correlation was only seen in the ML form (*r* = 0.952 p = 0.003) (Fig 7D); and, 5) *TLR9*^+^ *versus IL-10*^+^: a significant positive correlation was only seen in the MLform (*r* = 0.828 p = 0.04) (Fig 7E).

## Discussion

This is the first study undertaken in Brazil (more precisely, in the Brazilian Amazon) that analyzed the involvement of *TLR*2, 4, and 9 within the context of the entire clinical-immunopathological spectrum of ACL caused by the two *Leishmania* species with the greatest pathogenic potential for humans [*L*. *(V*.*) braziliensis* and *L*. *(L*.*) amazonensis*] and their relationship with CD4^+^ and CD8^+^ T-cells, and TNF-α^+^, IL-10^+^ and TGF-β^+^ cytokines, aiming to better understand the immunopathogenesis and/or the immunopathology of that disease.

Although the roles of *TLR*2, 4, and 9 were the major interest of the present work, we could not fail to also consider the importance of other immunological factors, such as CD4^+^ and CD8^+^ T-cells, and the TNF-α^+^, IL-10^+^ and TGF-β^+^ cytokines that take part in the T-cell immune responses against ACL. In this way, despite there have been some earlier research concerning the participation of CD4^+^ and CD8^+^ T-cells in the immunopathogenesis of ACL, no agreement has existed about what cell types are most well-represented in the cell infiltrates found in cutaneous lesions of that disease [26–32]. Thus, in the present work, as well as in prior research by our group [3, 19], a higher expression of CD8^+^ over CD4^+^ T-cells was observed not only in the central LCL forms caused by *L*. (*V*.)*braziliensis* or *L*. (*L*.) *amazonensis*, but also in the other two forms caused by *L*. (*L*.) *amazonensis* (BDCL and ADCL). This confirms the greater prevalence of CD8^+^ T-cells in most clinical forms of the disease spectrum (LCL/*L*.*b* or *L*.*a*, BDCL/*L*.*a* and ADCL/*L*.*a*), with exception of the BDCL and ML forms caused by *L*. *(V*.*)braziliensis*—therefore reinforcing the likely dual role of CD8^+^T-cells in the immunopathology of ACL: i) cytotoxic effects via NLRP3 inflammation activation or IL-1β production during infection by *L*. *(V*.*)braziliensis* [33–36], or ii) a regulatory effect through the production of TGF-β and IL-10 cytokines in the presence of high parasite loads during infection by *L*. *(L*.*) amazonensis* [14]. There was also a greater expression of CD4^+^ T-cells in the polar ML/*L*.*b* form (hyper-reactivity cellular pole), precisely the clinical form with the highest expression of DTH^(++++)^ and T-cell reactivity, as well as with significant expressions of IFN-γ^+^ and TNF-α^+^, characterizing a typical CD4^+^/Th1-type immune response [3, 4]. Additionally, there were low expressions of both CD4^+^ and CD8^+^T-cells in the polar ADCL/*L*.*a* form (hypo-reactivity cellular pole), although with high expression of CD8^+^T-cells (with twice the expression of CD4^+^T-cells), which reinforces the determinant role of the CD4^+^T-cell response in the definition of T-cell immune response profile against infection, whether of resistance (CD4^+^/Th1) or susceptibility (CD4^+^/Th2) to infection. This certainly influences ACL evolution and may explain the development of clinical polar forms of that disease, such as ML/*L*.*b* (with a typical CD4^+^/Th1-type immune response) and ADCL/*L*.*a* (with typical CD4^+^/Th2-type immune response) [3, 4, 6]. Summarizing then, CD8^+^ T-cells appear to play dual role in the immunopathology of ACL depending on the antigenic environment where those cells are interacting, i.e., in cases of infection by *L*. *(V*.*)braziliensis* their cytotoxic action (via NLRP3 inflammasome activation or IL-1β production) may result in exacerbation of pathology [35, 36], or in cases of infection by *L*. *(L*.*) amazonensis*their regulatory action (via TGF-β and IL-10) may promote dissemination of infection [14].

With regards to the involvement of pro-inflammatory TNF-α^+^ and the two regulatory TGF-β^+^ and IL-10^+^ cytokines, the almost dichotomous behavior of these cytokines within the spectrum of ACL caused by *L*. (*V*.) *braziliensis* and *L*. (*L*.) *amazonensis* should be highlighted here, with TNF-α^+^expression shown to be strongly associated with clinical forms caused by *L*. *(V*.*) braziliensis* (principally the central LCL and polar ML), while TGF-β^+^andIL-10^+^ expressions demonstrated strong association with clinical forms caused by *L*. (*L*.) *amazonensis* (the central LCL, but principally the subpolar BDCL and polar ADCL). Reinforcing this dichotomous character, a greater TNF-α^+^ expression was noted in the clinical forms caused by *L*. (*V*.) *braziliensis* (especially in the LCL and ML forms) as compared to TGF-β^+^andIL-10^+^ expressions (with exception of the BDCL form, where TGF-β^+^andIL-10^+^ expressions were greater). Similarly, TGF-β^+^andIL-10^+^ expressions in the clinical forms caused by *L*. (*L*.) *amazonensis* (LCL, BDCL, and ADCL) were always greater than TNF-α^+^ expression. Those results therefore represent strong evidence of the ability of those two *Leishmania* species [*L*. (*V*.) *braziliensis and L*. (*L*.) *amazonensis*] to modulate T-cell immune responses in ACL to opposite immunopathogenic poles (such as CD4^+^/Th1 [*L*. (*V*.)*braziliensis*] or CD4^+^/Th2 [*L*. (*L*.) *amazonensis*]) [4–6]. Within that context, the only exceptional situation arises in terms of the BDCL form caused by *L*. (*V*.)*braziliensis*, in which TNF-α^+^expression was lower than TGF-β^+^ or IL-10^+^ expression–exactly in the clinical form that occurs with partial inhibition of the T-cell immune response (DTH^**+/-**^), thus facilitating infection dissemination [3, 19]. This suggests that in individuals with less immunological competence (CD4^+^/Th1<Th2) and/or who are overloaded with infectious metacyclic promastigotes forms, the parasite can escape from the protective immune response and multiply in an uncontrolled fashion and disseminates to other skin areas and/or the nasopharyngeal mucosa [4].

Based on the above comments, the generalization of the functional roles of some cytokines, such as the TNF-α^+^, must be considered with a certain caution as it is generally regarded as a pro-inflammatory cytokine with a pivotal role in macrophage activation and parasite control [37, 38]. In that respect, it should be mentioned that the use of TNF-α^+^ antagonists in asymptomatic individuals can promote the reactivation of infection by *Leishmania* sp. and the emergence of clinical signs of leishmaniasis–thus confirming the protective role of TNF-α^+^ [39, 40]. However, when TNF-α^+^ expression was examined within the context of ACL caused by *L*. *(V*.*)braziliensis* or *L*. *(L*.*) amazonensis*, a weak expression of that cytokine on the associated spectrum side with *L*. *(L*.*) amazonensis* was noted; a strong TNF-α^+^expression was noted, however, in terms of the disease spectrum associated with *L*. *(V*.*)braziliensis*. This suggests that the functional activity of that cytokine is significantly influenced by its antigenic environment–that is, the parasite species involved [*L*. *(V*.*)braziliensis* or *L*. *(L*.*) amazonensis*]. Similarly, the two regulatory cytokines (TGF-β^+^andIL-10^+^) demonstrated strong expression in terms of the infection spectrum caused by *L*. (*L*.) *amazonensis* but weak expression in terms of the infection spectrum caused by *L*. *(V*.*)braziliensis*. This indicates, once again, that the antigenic environment exercises a crucial influence on the functional activities of those cytokines. Corroborating those results, recent work showing a lesser IL-10^+^expression in the central LCL form caused by *L*. *(V*.*)braziliensis* than in the same LCL form caused by *Leishmania (V*.*)* spp. should be mentioned–for it indicates that the functional activity of that cytokine can be differentially influenced in the same clinical form (LCL) caused by different *Leishmania* species of the same subgenus [24].

In considering the roles of *TLR*2, 4, and 9, it is important to stress that large numbers of papers related to the roles of *RTLs* in *Leishmania*-infection have been based on *in vitro* or experimental mouse models, using principally Old World *Leishmania* species such as *L*. *(L*.*) major*, *L*. *(L*.*) donovani*, and *L*. *(L*.*) tropica* [41–46]. The roles of *TLR*2, 4, and 9 in the clinical-immunopathological spectrum of ACL caused by New World species [specifically *L*. *(V*.*) braziliensis* and *L*. *(L*.*) amazonensis* from the Brazilian Amazon], however, have not yet been described.

We demonstrated here that *TLR*2, 4, and 9 are present in all forms of the clinical-immunopathological spectrum of ACL, although with a stronger interaction of *TLR*2 and 4 with *L*. *(V*.*) braziliensis*, as indicated by the greater expression of those receptors in the LCL and BDCL forms of the disease associated with that species. On the other hand, there appears to be a strong interaction of *TLR*9 with *L*. *(L*.*) amazonensis*, in light of its greater expression in the ADCL and BDCL forms; there is also significant expression of that receptor in the BDCL form in response to *L*. *(V*.*) braziliensis*. Based on those analyses, and their similarity to observations concerning the expressions of the TNF-α^+^, TGF-β^+^, and IL-10^+^ cytokines, there are also almost dichotomous expressions of *TLR*2, 4, and 9 in the clinical-immunopathological spectrum of ACL caused by *L*. *(V*.*) braziliensis* and by *L*. *(L*.*) amazonensis*, suggesting the possibility that specific antigens of those *Leishmania* species may be influencing the modulation of the innate immune response against infection.

In that respect, some *in vitro* studies have indicated that the interactions of *TLRs* with different *Leishmania* species may lead to different immune responses [47]. Those studies have shown that *TLR*2 is activated by glycolipids present in the cell membranes of *L*. *(L*.*) major*, resulting in macrophage stimulation to secrete cytokines such as IL-12 and TNF-α. During infection by *L*. *(L*.*) donovani*, on the other hand, p38 MAPK activation is mediated by *TLR*2, lowering IL-12 production while increasing IL-10 production [48]. Those divergent immune responses related to *in vitro* interactions of *TLR*2 in those two *Leishmania* species [*L*. *(L*.*) major* and *L*. *(L*.*) donovani*] indicated support for the results of the present analysis with clinical samples of ACL caused by *L*. *(V*.*) braziliensis* and *L*. *(L*.*) amazonensis*—analyses that indicated greater expression of *TLR*2 and 4 within the clinical forms caused by *L*. *(V*.*) braziliensis*. In that way, our results also revealed a high expression of TFN-α^+^ which corroborates previous evidence indicating that the immune response on that side of the disease spectrum is predominantly of the CD4^+^/Th1-type [3, 4]. It should also be emphasized that the negative correlation between *TLR*4^+^ and TNF-α^+^ in the polar ML form caused by *L*. *(V*.*) braziliensis* appears to reflect the strong expression of the CD4^+^/Th1-type immune response characteristic of that form of the disease.

The limited expression of *TLR*2 and 4 on the disease spectrum pole relative to clinical forms caused by *L*. *(L*.*) amazonensis* (which showed strong TGF-β^+^ and IL-10^+^ expressions) appears to demonstrate that the immune response in that direction has a clear CD4^+^/Th2-type profile [3, 4]. As such, and based on previous observations indicating that the parasites possess mechanisms capable of modulating the inflammatory responses induced by *TLRs* [47, 48], the concept presented here is that the interactions of *TLR*2 and 4 with *L*. *(V*.*) braziliensis* seem to be related to a beneficial T-cell immune response. Corroborating that interpretation, the negative correlation between *TLR*2^+^andTGF-β^+^ in the central LCL form caused by *L*. *(V*.*) braziliensis* suggests a likely protector effect of *TLR*2^+^ by inhibiting TGF-β^+^.

It should also be noted that *TLR*2 and 4 have been considered pivotal actors in the immune response of ACL, as it has been shown in *in vitro* trials that infections caused by *L*. *(V*.*) braziliensis* and *L*. *(V*.*) panamensis* induce increased expression of those macrophage receptors in humans–indicating their participation in the innate immune response to ACL caused by those *Leishmania* species [49]. Similarly, and corroborating the results of the present work, it has been demonstrated that *TLR*2 expression in cutaneous lesions of patients with LCL caused by *L*. *(V*.*) braziliensis* is greater than that observed in healthy individuals, suggesting the importance of that receptor in immunopathogenesis [13].

Once the differential expressions of *TLR*2 and 4 in the two different *Leishmania* species examined [*L*. *(V*.*) braziliensis* and *L*. *(L*.*) amazonensis*] have been established, it becomes necessary try to explain those differences. A number of factors can interfere with *TLR* expression on cell surfaces [50, 51]. Some workers have suggested that factors related to the parasites themselves [such as the lipophosphoglycan (LPG) molecule (the principal antagonist of *TLR*2 and 4 and responsible for more than 95% of the surface composition of *Leishmania*)] have crucial roles in the initial events of *Leishmania*-macrophage interactions [41]. Ibraim et al. [52], for example, in evaluating the role of *L*. *(V*.*) braziliensis*-LPG in the immune response of ACL, determined that it not only stimulates messenger RNA transcription (thus promoting increased *TLR*2 expression) but also induces NF-kB translocation (responsible for inducing the expression of pro-inflammatory cytokines)–resulting in NO expression, as well as IFN-γ and TNF-α cytokine induction. That induction is mediated by linking *L*. *(V*.*) braziliensis*-LPG to *TLR*2, indicating the crucial role of that receptor in innate defenses against infection.

Certain species of *Leishmania*, on the other hand, can subvert the activation of *TLR* pathways, which may, in turn, be related to their capacity to inhibit NF-κB activation and interfere with the signaling functions of those receptors [50]. Cameron et al. [53] demonstrated that *L*. *(L*.*) mexicana*-amastigote form (a species belonging to the same subgenus as *L*. *(L*.*) amazonensis* and the causal agent of LCL and ADCL forms in Mexico) possesses enzymes with proteolytic actions against NF-kB that can impede the final reaction step in the activation cascade of *TLRs*–leading to the repression of IL-12 production. In this case, *L*. *(L*.*) mexicana* increases its production of those peptidases after phagocytosis in order to reduce inflammatory responses and allow its own multiplication [54]. Similar results were reported by Shweash et al. [55], who demonstrated that *L*. *(L*.*) mexicana* inhibits IL-12 production through *TLR*4 –characterizing a subversion of CD4^+^/Th1-type protective immune response.

Specifically in terms of *L*. *(L*.*) amazonensis*, Nogueira et al. [56] demonstrated by *in vitro* experiments that a parasite strain originating from a clinical case of ADCL was capable of activating *TLR*4, and that this activation was associated with LPG of the parasite. We demonstrated here that *TLR*4 was observed not only in the ADCL form but also in the other clinical forms (LCL and BDCL) caused by *L*. *(L*.*) amazonensis*, although without differences in its expression. It was moderately expressed in the central LCL form but weakly expressed in the subpolar BDCL and polar ADCL forms, an observation that could be associated with its persistent stimulation by the parasite, as both the BDCL and ADCL clinical forms are characterized by elevated parasite loads in macrophages–resulting in the saturation of *TLR*4 activity or its gene expression pathway due to limitations of messenger RNA transcription. That situation has been described in other models of *TLR* activation under conditions of prolonged exposure to lipopolysaccharide/LPS and lipoteichoic acid/ALT (bacterial PAMPs) that diminish *TLR*2 expression on the surfaces of mesenchymal cells [57].

Little has been observed in terms of the role of *TLR*9 in ACL, with exception of a single publication demonstrating that the expression of that receptor was associated with granuloma in the LCL form caused by *L*. *(V*.*) braziliensis* [16]. According to Abou Faker et al. [58], *TLR*9 has an important role in the innate immune response due to its activation of NK cells and macrophages through the production of IL-12. *TLR*9 expression was observed in the present work in all forms of the clinical-immunopathological spectrum of ACL, although in significantly more expressive manners in the clinical forms associated with *L*. *(L*.*) amazonensis*, principally when T-cell immune response was clearly suppressed, as in ADCL (CD4^+^/Th2, DTH^-^) or BDCL (CD4^+^/Th1≥Th2; DTH^-^). This leads us to believe that, different from reports of *TLR*9 associated with the presence of granuloma in patients with the LCL form caused by *L*. *(V*.*) braziliensis* [16], *TLR*9 is associated with a susceptibility profile to ACL because its significant expression on the side of the spectrum associated with *L*. *(V*.*) braziliensis* was only observed in the BDCL clinical form, which is also associated with a partially suppressed T-cell immune response (CD4^+^Th1≥Th2; DTH^**+/-**^) [3, 4, 6, 19]. It has also recently been demonstrated that *L*. *(L*.*) amazonensis* promotes its own survival by inducing the expression of CD200, an immunoregulatory molecule thatinhibits macrophage activation [59]. This same situation was observed in*TLR*2-/-, *TLR*3-/- and *TLR* 4-/- macrophages, but not in MyD88-/-, TRIF-/- and *TLR*9-/- macrophages. When those macrophages were treated with *TLR* agonists a strong increase in *TLR*9 activation was observed, suggesting that signaling mediated by *TLR*9 is required to induce CD200 by *L*. *(L*.*) amazonensis*[Sauter I, personal communication]. That may explain the higher interaction of *TLR*9 with the side of the spectrum associated with *L*. *(L*.*) amazonensis*, principally in those BDCL and ADCL clinical forms.

While the current analysis yielded quite satisfactory results, an interesting and somewhat surprising finding must be noted that is related to the fact that the greatest cellular reactivity with the ML form in the clinical-immunopathological spectrum of ACL caused by *L*. *(V*.*) braziliensis* demonstrated the lowest expressions of *TLR*2, 4, and 9. This seems to reflect the characteristics of the predominantly lymphoplasmacytic cellular infiltrate present in that form of the disease, with wide numerical supremacy over the macrophages that serve as target cells to be double-marked by the immunohistochemistry of *TLR*2, 4, and 9 (CD68 +*TLR*2, 4, and 9). It is therefore possible that this immunohistochemical strategy may have been influenced the weak expression of those receptors in ML form of the disease. While that clinical form shows low *TLR* expression, a positive correlation between *TLR*4 and 9 with IL-10^+^ was noted, which corroborates the weak IL-10^+^ expression in the ML/*L*.*b* form–which, as was described above, is characterized by strong CD4^+^/Th1-type immune response and significant IFN-γ and TNF-α expressions [3, 5, 6].

## Supporting information

S1 Table Antibodies used in the immunohistochemical analyses. (PDF)

S1 Fig CD4^+^ T-cell density in the different clinical forms of ACL caused by *L*. (*V*.) *braziliensis* (*L*.*b*)and *L*. (*L*.) *amazonensis* (*L*.*a*). (*): p< 0.05; (⊢⊣): significant differences found between anergic diffuse cutaneous leishmaniasis (ADCL/*L*.*a*) and mucosal leishmaniasis (ML/*L*.*b*), compared with the other clinical forms.
(TIF)

S2 Fig CD8^+^ cell densities in the different clinical forms of ACL caused by *L*. (*V*.) *braziliensis (L*.*b)* and *L*. *(L*.*) amazonensis* (*L*.*a*). (*):p< 0.05; (⊢⊣): significant differences found between anergic diffuse cutaneous leishmaniasis (ADCL/*L*.*a*) compared with the other clinical forms.
(TIF)

S3 Fig TNFα^+^ cell density expressions in the different clinical forms of ACL caused by *L*. *(V*.*) braziliensis*(*L*.*b*)and *L*. *(L*.*) amazonensis* (*L*.*a*). (*) = p< 0.05. (⊢⊣) = significant differences found between anergic mucosal leishmaniasis (ML/*L*.*b*) compared with the other clinical forms.
(TIF)

S4 Fig TGF-β^+^ cell densities in the different clinical forms of ACL caused by *L*. (*V*.) *braziliensis* (*L*.*b*)and *L*. (*L*.) *amazonensis* (*L*.*a*). (*) = p< 0.05. (⊢⊣) = significant differences found between anergic diffuse cutaneous leishmaniasis (ADCL/*L*.*a*) compared with the other clinical forms.
(TIF)

S5 Fig IL-10^+^ cell densities in the different clinical forms of ACL caused by *L*. (*V*.) *braziliensis* (*L*.*b*)and *L*. (*L*.) *amazonensis* (*L*.*a*). (*) = p <0.05. (⊢⊣) = significant differences between anergic diffuse cutaneous leishmaniasis (ADCL/*L*.*a*) and mucosal leishmaniasis (ML/*L*.*b*), with the other clinical forms.
(TIF)
