# Supplementary material for: Toll-like receptors 2, 4, and 9 expressions over the entire clinical and immunopathological spectrum of American cutaneous leishmaniasis due to Leishmania (V.) braziliensis and Leishmania (L.) amazonensis
Source: PLoS One. 2018 Mar 15;13(3):e0194383. doi: 10.1371/journal.pone.0194383 (PMC5854399; doi:10.1371/journal.pone.0194383)
Supplement: S1 Table — (PDF) [file pone.0194383.s001.pdf]

**Antibodies**	**Dilution**	**Label**	**Code**	**Origin**
Anti human CD68	1: 50	DAKO	PMG1	Mouse
Anti human CD4	1:	DAKO	M7310	Mouse
Anti human CD8	1	DAKO	M7103	Mouse
Anti-TNF-α	1:	R&D	AF210NA	Mouse
Anti-TGF-β	1:	SANTA CRUZ	SC146	Mouse
Anti-IL-10	1:400	ABCAM	Ab34843	Mouse
Anti-*Leishmania*	1:1000	*in house*	-	Mouse
TLR-2	1: 50	SANTA CRUZ	SC8689	Goat
TLR-4	1: 50	SANTA CRUZ	SC8694	Goat
TLR-9	1: 100	SANTA CRUZ	SC13125	Goat
