# Supplementary figures and images for: Toll-like receptors 2, 4, and 9 expressions over the entire clinical and immunopathological spectrum of American cutaneous leishmaniasis due to Leishmania (V.) braziliensis and Leishmania (L.) amazonensis
Source: PLoS One. 2018 Mar 15;13(3):e0194383. doi: 10.1371/journal.pone.0194383 (PMC5854399; doi:10.1371/journal.pone.0194383)

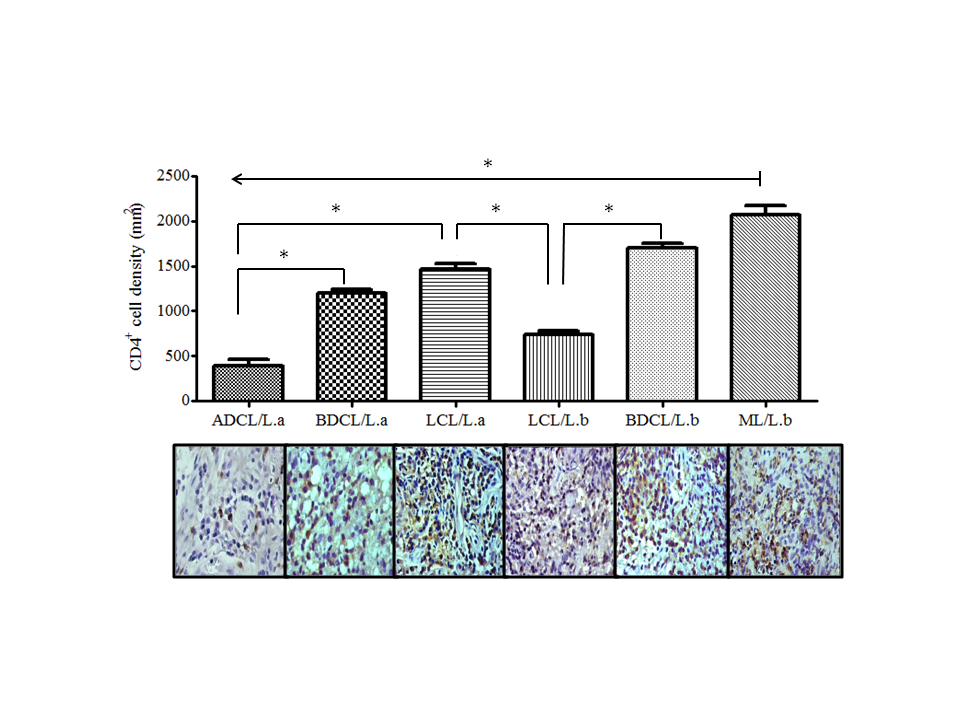

Supplement: S1 Fig — (*): p< 0.05; (⊢⊣): significant differences found between anergic diffuse cutaneous leishmaniasis (ADCL/L.a) and mucosal leishmaniasis (ML/L.b), compared with the other clinical forms. (TIF) [file pone.0194383.s002.tif]

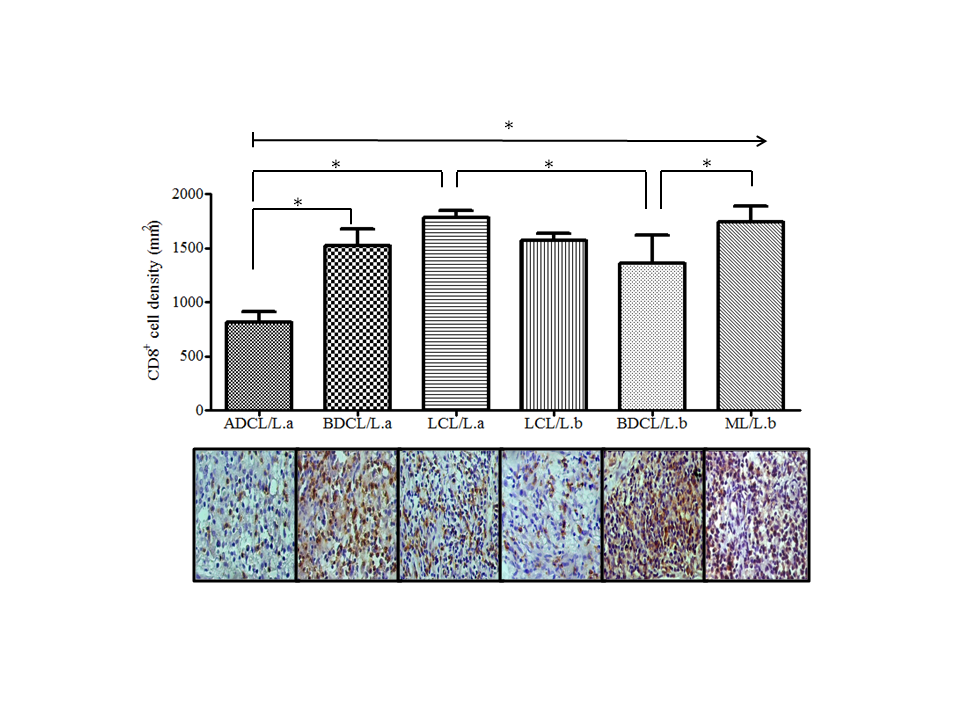

Supplement: S2 Fig — (*):p< 0.05; (⊢⊣): significant differences found between anergic diffuse cutaneous leishmaniasis (ADCL/L.a) compared with the other clinical forms. (TIF) [file pone.0194383.s003.tif]

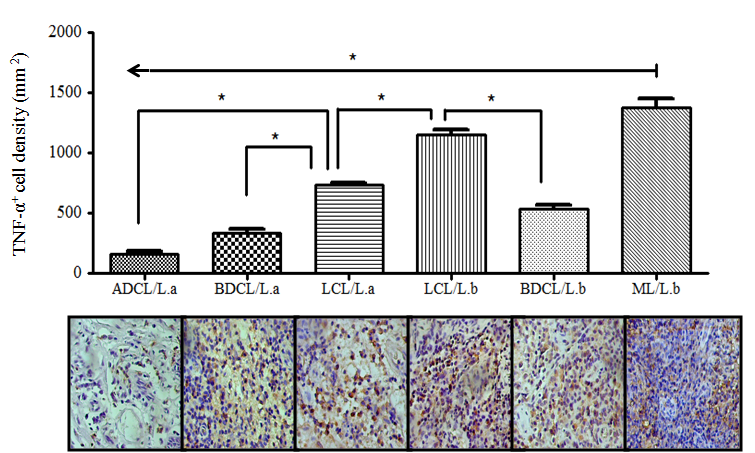

Supplement: S3 Fig — (*) = p< 0.05. (⊢⊣) = significant differences found between anergic mucosal leishmaniasis (ML/L.b) compared with the other clinical forms. (TIF) [file pone.0194383.s004.tif]

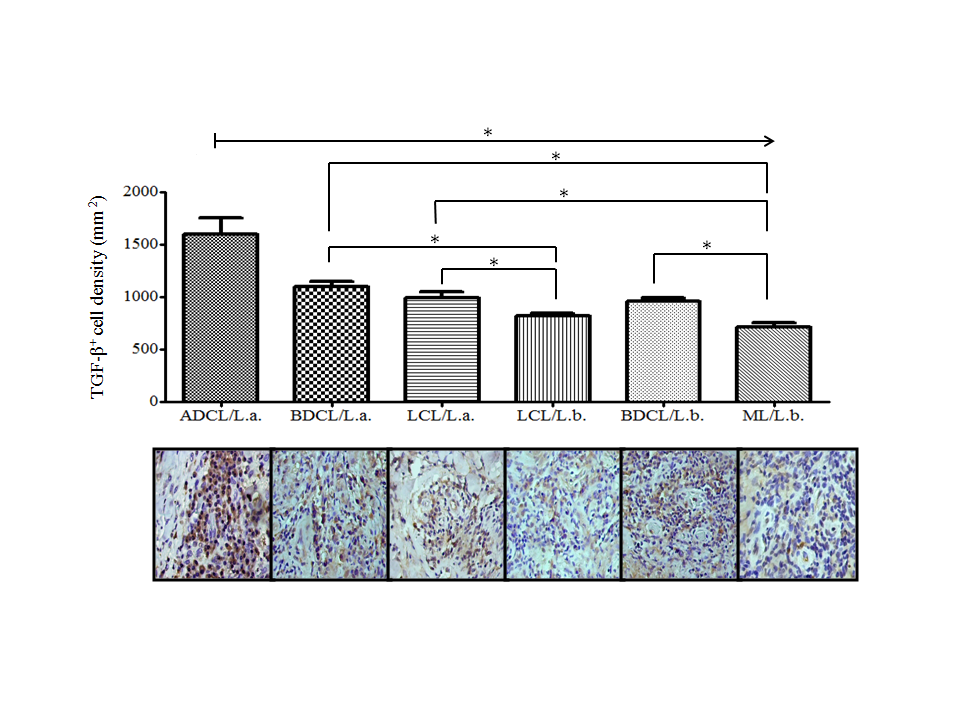

Supplement: S4 Fig — (*) = p< 0.05. (⊢⊣) = significant differences found between anergic diffuse cutaneous leishmaniasis (ADCL/L.a) compared with the other clinical forms. (TIF) [file pone.0194383.s005.tif]

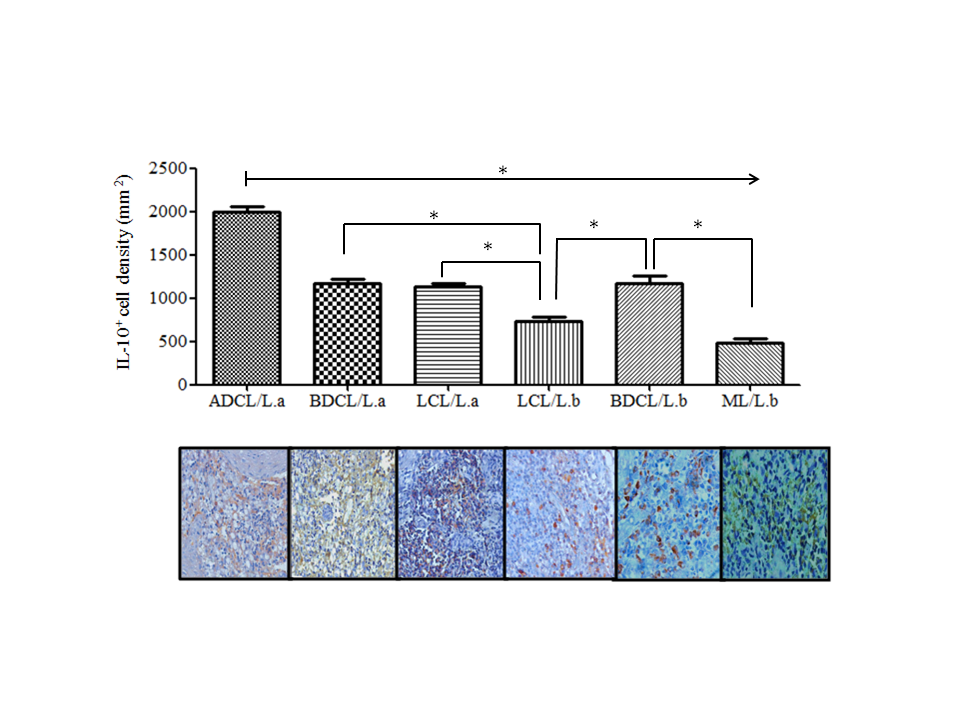

Supplement: S5 Fig — (*) = p <0.05. (⊢⊣) = significant differences between anergic diffuse cutaneous leishmaniasis (ADCL/L.a) and mucosal leishmaniasis (ML/L.b), with the other clinical forms. (TIF) [file pone.0194383.s006.tif]
